# Imaging biomarkers in neurodegeneration: current and future practices

**DOI:** 10.1186/s13195-020-00612-7

**Published:** 2020-04-27

**Authors:** Peter N. E. Young, Mar Estarellas, Emma Coomans, Meera Srikrishna, Helen Beaumont, Anne Maass, Ashwin V. Venkataraman, Rikki Lissaman, Daniel Jiménez, Matthew J. Betts, Eimear McGlinchey, David Berron, Antoinette O’Connor, Nick C. Fox, Joana B. Pereira, William Jagust, Stephen F. Carter, Ross W. Paterson, Michael Schöll

**Affiliations:** 1 Wallenberg Centre for Molecular and Translational Medicine and the Department of Psychiatry and Neurochemistry, University of Gothenburg, Sahlgrenska University Hospital, Gothenburg, Sweden; 2 Centre for Medical Image Computing (CMIC), Department of Computer Science & Department of Medical Physics and Biomedical Engineering, University College London, London, UK; 3 Department of Radiology and Nuclear Medicine, Amsterdam Neuroscience, Vrije Universiteit Amsterdam, Amsterdam UMC, Amsterdam, Netherlands; 4 Neuroscience and Aphasia Research Unit, Division of Neuroscience and Experimental Psychology, The University of Manchester, Manchester, UK; 5 German Center for Neurodegenerative Diseases (DZNE), Magdeburg, Germany; 6 Division of Brain Sciences, Imperial College London, London, UK; 7 United Kingdom Dementia Research Institute, Imperial College London, London, UK; 8 Cardiff University Brain Research Imaging Centre (CUBRIC), School of Psychology, Cardiff, UK; 9 Dementia Research Centre, UCL Institute of Neurology, University College London, London, UK; 10 Department of Neurological Sciences, Faculty of Medicine, University of Chile, Santiago, Chile; 11 Institute of Cognitive Neurology and Dementia Research, Otto-von-Guericke-University Magdeburg, Magdeburg, Germany; 12 Trinity College Dublin, The University of Dublin, Dublin, Ireland; 13 Clinical Memory Research Unit, Department of Clinical Sciences Malmö, Lund University, Lund, Sweden; 14 Division of Clinical Geriatrics, Department of Neurobiology, Care Sciences and Society, Karolinska Institutet, Stockholm, Sweden; 15 Helen Wills Neuroscience Institute, University of California, Berkeley, USA; 16 Molecular Biophysics and Integrated Bioimaging, Lawrence Berkeley National Laboratory, Berkeley, CA USA; 17 Department of Psychiatry, School of Clinical Medicine, University of Cambridge, Cambridge, UK; 18 Wolfson Molecular Imaging Centre, Division of Neuroscience and Experimental Psychology, MAHSC, University of Manchester, Manchester, UK; 19 Department of Clinical Physiology, Sahlgrenska University Hospital, Gothenburg, Sweden

**Keywords:** Neurodegenerative diseases, Neuroimaging, PET, MRI, Alzheimer’s disease, Machine learning, dementia

## Abstract

There is an increasing role for biological markers (biomarkers) in the understanding and diagnosis of neurodegenerative disorders. The application of imaging biomarkers specifically for the in vivo investigation of neurodegenerative disorders has increased substantially over the past decades and continues to provide further benefits both to the diagnosis and understanding of these diseases. This review forms part of a series of articles which stem from the University College London/University of Gothenburg course “Biomarkers in neurodegenerative diseases”. In this review, we focus on neuroimaging, specifically positron emission tomography (PET) and magnetic resonance imaging (MRI), giving an overview of the current established practices clinically and in research as well as new techniques being developed. We will also discuss the use of machine learning (ML) techniques within these fields to provide additional insights to early diagnosis and multimodal analysis.

## Introduction

Neurodegenerative diseases, including Alzheimer’s disease (AD), are now recognised to start years before symptoms appear [1]. Studies of the genetically caused familial AD have proposed a sequence of pathologic events, starting with build-up and accumulation of amyloid-β (Aβ), that can now be measured in the brain (using positron emission tomography (PET) imaging) and in cerebrospinal fluid (via lumbar puncture) and ending with cognitive deficits and dementia [2]. These events appear to start demonstrating abnormalities in a distinct order, but also overlap temporally.

Specifically considering AD, the National Institute on Ageing and Alzheimer’s Association (NIA-AA) has developed the research framework for the diagnosis of AD [3]. This categorises diagnoses into the AT(N) scale, referring to Aβ, tau and neurodegeneration. These three pathologies can all be (spatially and temporally) identified in vivo with current imaging biomarkers. Further biomarkers that could be added to contribute to the ATN categories (as discussed in detail in [3]) could come from the imaging modalities discussed in this review. Further refinement of diagnostic cut-offs for each of these imaging-derived biomarkers will then also provide increases in the sensitivity and specificity for the respective modality.

Neuroimaging has become a standard tool in the clinical work up of individuals suspected of having a neurodegenerative disease. The use of various magnetic resonance imaging (MRI) techniques and the development of novel PET ligands have led to the ability to understand these diseases in vivo like never before. Access to these tools has provided access to a plethora of objective measures which can indicate both the presence and progression of these diseases. This is useful for patients in a clinical setting but can also be used for the targeted recruitment to clinical treatment trials and tracking of any treatments that are undergoing clinical trial both in terms of efficacy of treatment but also for safety monitoring. Neurodegenerative disorders are increasingly requiring the input of multiple disciplines for both the diagnosis and understanding of these diseases, and imaging biomarkers have a role to play in the wider collaborative approach to understanding these diseases as well.

There have been considerable advances in the portfolio of PET ligands available for use in identifying biomarkers associated with neurodegeneration, some of which have progressed to use in the clinic and others present promising new avenues for understanding these neurodegenerative diseases. MRI techniques are also being used to help with both the diagnosis and the development of our understanding, with structural MRI still being the most widely available imaging tool for neurodegeneration. This review article will give a brief overview of the established and upcoming practices both within the PET and MRI fields in relation to neurodegeneration as well as how machine learning can be an aid to these modalities.

## Positron Emission Tomography

### [18F]FDG PET

[18F]-2-Fluoro-2-deoxy-d-glucose (FDG) was first introduced as a PET tracer for neuroimaging in 1979 [4] and has since been established as a routine research and clinical biomarker for diagnosing dementia [5]. Glucose is the brain’s main source of energy. It circulates in the blood and crosses the blood-brain barrier. When energy is needed, glucose is phosphorylated as the first step of energy being made available. FDG is an artificial analogue of glucose, which mimics glucose’s action until it is phosphorylated. Phosphorylated FDG gets trapped in tissue and is not metabolised further. The rate of FDG trapping is proportional to glucose metabolism. Rocher et al. showed that regional glucose consumption is related to synaptic activity [6], and decreased regional FDG trapping (hypometabolism) is interpreted as a sign of synaptic and neuronal damage.

Protocols for acquiring FDG images can vary between sites. After intravenous injection of FDG into a fasting subject (fasted for ~ 4 h), and waiting a minimum of 30 min to allow FDG to circulate, PET data is acquired, typically for 10–30 min. Absolute glucose metabolism can be calculated by using an arterial input function derived from arterial blood, but more commonly the standardised uptake value (SUV) is calculated using body mass (kg) and injected dose of FDG (MBq). Regional SUV ratios (SUVRs) can be generated using a standard reference region, normally a region unaffected by the disease process, e.g. the grey matter of the cerebellum in AD [7].

Clinical application of FDG PET varies between countries, but regional neocortical hypometabolism is accepted to be useful to help differentiate dementias even though regional patterns can overlap [8–10]. In AD, hypometabolism can appear before visible atrophy [11] and a symmetrical hypometabolism in the temporoparietal, posterior cingulate and medial temporal cortices is usually seen. Reported sensitivity and specificity for AD diagnosis vary from study to study, but in 2015, Smailagic et al. [12] found the sensitivity for conversion from mild cognitive impairment (MCI) to AD was 76% at 82% specificity. In frontotemporal dementia, hypometabolic regions include the frontal and anterior temporal lobes, cingulate gyri, uncus, insula, basal ganglia and medial thalamus. The hypometabolism is often asymmetric [13] with sensitivity of 88% and specificity of 91%. Occipital hypometabolism occurs in both posterior cortical atrophy (an atypical form of AD) and dementia with Lewy bodies (DLB) [14]: dopamine transporter imaging could be used to differentiate these dementia types since dopamine transport is decreased in DLB.

While useful, FDG PET has limitations. Decreased uptake can be caused by a diminished cerebrovascular circulation or by metabolic disorders such as diabetes rather than decreased synaptic activity [15]. As the scan involves radiation exposure, it is not recommended to be repeated more frequently than annually. The PET process itself (isotope production, radiochemistry, scan) is expensive compared to MRI, and [18F] has a short half-life of just under 2 h (110 min), which adds time pressure to scans. In summary, FDG PET is a useful biomarker for investigating neuronal injury in dementia.

### Amyloid-β PET

The involvement of Aβ in the pathological expression of AD has been known for over 25 years [16]. This involves the aggregation of fibrillar Aβ causing the creation of so-called Aβ plaques in the brain [17]. The current hypothesis is that plaque accumulation induces multiple downstream alterations that lead to neurodegeneration and cognitive decline. Our understanding of these downstream alterations has changed over the years and now includes not only inflammation but synaptic alterations, functional changes and alterations in tau [18, 19]. Historically, the only way to definitively classify a person as having AD was through post mortem examination of their brain tissue for Aβ plaques and neurofibrillary tangles (NFTs) predominantly consisting of hyperphosphorylated tau protein. With the advent of Aβ PET tracers, it is now possible to have an in vivo, quantifiable measure of a key biomarker of AD, thereby allowing a possible diagnosis much earlier [20]. Aβ in the brain displays a pattern of deposition that begins in medial frontal and basal temporal areas, progressing to include the neocortex, primary sensory-motor areas and finally the MTL and striatum [21–23].

The first Aβ-specific PET tracer introduced to provide accurate imaging data of Aβ pathology is Pittsburgh compound B (PiB) labelled with C-11 [24, 25]. The compound is derived from thioflavin-T and is known to have a high affinity to the Aβ plaques. This has resulted in its widespread use both as a diagnostic tool and as a reference for other Aβ PET tracers [25]. While [11C]PiB is useful in research settings, its widespread utility is limited by the short half-life of C-11 necessitating a local cyclotron and radiochemistry. Therefore, the development of an F-18 (110-min half-life)-based Aβ tracer was required for routine clinical use as those can be delivered from an off-site cyclotron. There are currently three F-18-labelled Aβ PET tracers approved by the FDA and EMA for clinical use. These are [18F]flutemetamol, [18F]florbetapir and [18F]florbetaben. These tracers have shown to behave similarly to PiB in head-to-head studies [26–28], and some have been verified histologically [29]. Another Aβ-specific tracer, [18F]NAV4694, is thought to overcome some of the reported shortcomings of the previous-generation tracers, mainly “off-target” binding [30]. The range of tracers being used, all with differing uptake characteristics and varying pharmacokinetics, means that care must be taken when performing analyses. Each of these tracers will require their own analysis pipeline with possible differences in reference regions [31, 32]. Cut points for Aβ positivity will also vary between tracers [33]. This is why there has been an ongoing development to standardise quantitative Aβ imaging measures using the “centiloid scale” [34]. Work is now ongoing to validate centiloid scaling between various tracers and against other biomarkers [35–37]. Standardisation such as centiloid scaling has yet to be applied in tau imaging to a greater extent [38].

As post mortem and in vivo biomarker evidence closely associate Aβ pathology with AD, Aβ PET tracers have become standard tools for clinicians to aid in their diagnosis of patients suspected of having AD [39]. These tracers also have a key place in AD research in differentiating diagnostic groups and tracking disease progression [40]. In addition, these tracers are readily used for the evaluation of therapeutic trial outcomes to examine the effects of drugs removing brain Aβ [41, 42].

### Tau PET

**Fig. 1:**
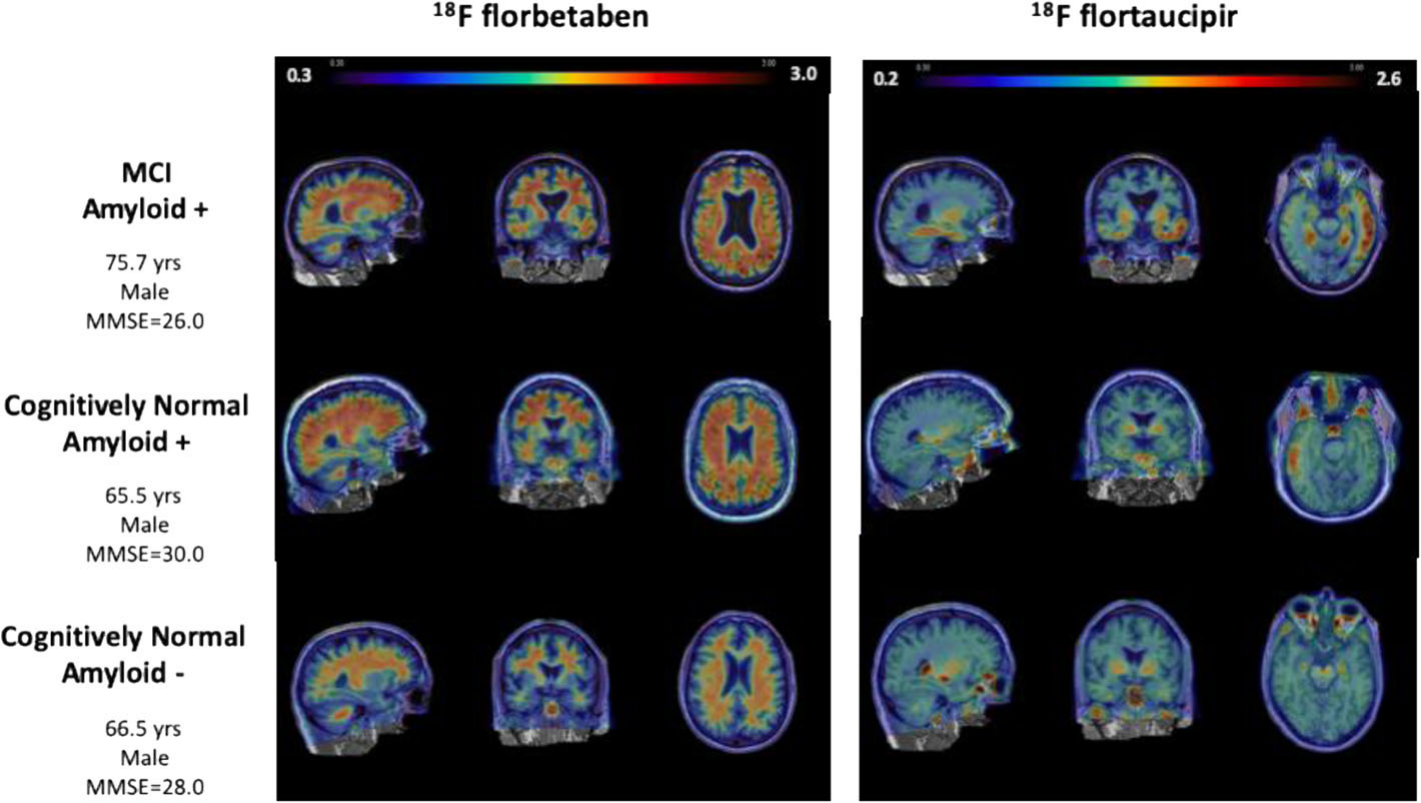
Comparison of [18F]florbetaben and [18F]flortaucipir for three patients. The authors would like to acknowledge Dr. Susan Landau (UC Berkeley) for her assistance in the creation of this figure. Scale is standardised uptake value ratio (SUVr)

The advent of tau-specific PET tracers has marked the beginning of a new era with potential applications in differential diagnosis and prognosis and serving as a secondary outcome measure for clinical trials [43]. Tau is physiologically involved in the stabilisation of microtubules and can present with three or four repeat (3R/4R) microtubule-binding domains [44, 45]. First-generation tau PET ligands all seem to bind mixed 3R/4R paired helical filament (PHF) formations of tau [46–50] and include [11C]PBB3, a series of “THK” tracers ([18F]THK523, [18F]THK5105 and [18F]THK5351), and [18F]flortaucipir (formerly [18F]T807 or [18F]AV1451) [51–53]. As these “first-generation” tracers face challenges such as off-target binding, novel tau compounds have been developed, though their relationship with clinical outcome measures has yet to be established in larger cohorts [54, 55]. Notable “second-generation” tau ligands include [18F]RO948, [18F]GTP1, [18F]PI2620, [18F]PM-PBB3 and [18F]MK6240 [54, 56–60], which have shown reduced off-target binding with similar on-target signal response [61, 62]. As currently available tau PET ligands bind AD-like mixed 3R/4R tau pathology, the utility of tau PET in pure 3R or 4R tauopathies, such as progressive supranuclear palsy and corticobasal degeneration, has shown to be less persuasive [57]. So far, tau PET studies in clinical settings have mostly been performed within the field of AD using the [18F]flortaucipir tracer (see Fig. 1).

In AD, tau PET imaging studies have demonstrated that tau deposition seems to follow the staging pattern revealed by Braak and Braak, suggesting tau spreads from the entorhinal cortex (Braak I/II) to the inferolateral temporal and medial parietal lobes (Braak III/IV) and finally the neocortex (Braak V/VI) [63–65]. This vulnerability of brain regions for tau pathology overlaps with brain regions underlying the different clinical phenotypes in typical and atypical AD dementia and correlates with atrophy and reduced glucose metabolism in those regions, a relationship that is not found with Aβ [66, 67]. Tau pathology in Braak I/II is also commonly observed in cognitively unimpaired controls, which likely reflects an age-related process of tau accumulation. Also, in these cognitively unimpaired individuals, tau seems to be associated with regional atrophy and hypometabolism, as well as to subtle cognitive deficits [68–73]. Furthermore, a recent longitudinal study has shown that both baseline and change in [18F]flortaucipir are related to changes in cognition [74]. Hence, both the amount and distribution of tau PET accurately reflect cognitive symptoms and deterioration. The clinical utility of tau PET has recently been demonstrated in a multi-centre study highlighting the ability of [18F]flortaucipir to discriminate between AD dementia and non-AD neurodegenerative diseases, with highest sensitivity (96.8%) and specificity (87.9%) using several thresholds applied to temporal and temporoparietal regions [75]. Furthermore, while tau PET and tau measured in the cerebrospinal fluid (CSF) performed equally well in separating prodromal AD from controls, tau PET outperformed tau-CSF in discriminating prodromal AD from AD dementia [76].

Although tau PET imaging shows great potential for implementation into the clinic, the high regional specificity of tau requires careful selection of regional and global measures for categorising individuals into tau-positive or tau-negative as suggested by the NIA-AA research framework. Different cut points for tau-tracer binding in different brain regions have been suggested, however, standardisation of such methodological aspects is needed. Furthermore, although the majority of AD patients present with both high Aβ and high tau burden, studies have shown that some AD patients present with high Aβ and low tau burden [77–79]. Possible explanations such as clinical misdiagnoses (with incidental Aβ co-pathology) or differences in tau conformations that might affect tracer binding are to be examined in future clinical tau PET studies and studies using novel tau PET ligands. Importantly, although the advent of tau PET tracers has greatly advanced our knowledge regarding the close relationship between tau pathology and downstream neurodegeneration events linked to cognitive decline, it remains as of yet unknown how Aβ relates to the development of tau, and how tau relates to the occurrence of neurodegeneration. A study by Jacobs and colleagues indicated that Aβ may facilitate the spread of tau from the medial temporal lobe to the downstream posterior cingulate cortex through the parahippocampal cingulum [80]. Ideally, multimodal longitudinal imaging studies are needed to elucidate the temporal relationships between pathology biomarkers.

### SV2A PET

In AD, Aβ and tau alongside neuroinflammation and vascular insufficiency lead to irreversible synaptic dysfunction and loss [81] causing the deleterious amnestic presentation of the disease. Loss of synapses and decreased synaptic density (particularly the vulnerable hippocampus/medial temporal lobe) are likely earlier events than neurodegeneration and important for drug targets. Previously, synaptic density changes could only be studied cross-sectionally from post mortem brain tissue or biopsy [82, 83]. Recently, these changes have been able to be visualised directly in vivo in humans [84].

PET ligands targeting the synaptic vesicle glycoprotein 2A (SV2A) form a potentially useful and exciting investigative tool to measure synapses [85]. SV2 is a 12 transmembrane domain integral protein with three isoforms (2A, 2B and 2C). SV2A is the most ubiquitous and is expressed as a transmembrane glycoprotein in secretory vesicles on presynaptic terminals. It is critical to synaptic function, particularly Ca2+-dependent exocytosis [86], and is known to be the binding site of levetiracetam [87].

[11C]UCB-J is a PET ligand developed to image SV2A with favourable brain uptake, kinetics and dosimetry shown in non-human primates [88]. There is a high correlation between in vitro SV2A UCB-J binding and in vitro synaptophysin density (*r*^2^ = 0.90 for GM regions) pre-clinically [4]. [11C]UCB-J has a high affinity for SV2A (Ki = 7 nm) and has been successfully displaced by levetiracetam in vivo in humans, with good dosimetry (4.5 mSv/MBq) [4]. Regional time-activity curves showed [11C]UCB-J has fast kinetics, was well described by a 1 tissue compartment model (1TC) or a simplified reference tissue model (SRTM) (reference centrum semi-ovale) and had a 3–9% mean test-retest variability in VT across regions [89].

Decreased [11C]UCB-J binding was first shown in a small disease group with temporal lobe epilepsy and mesial temporal sclerosis (*n* = 3), revealing region-specific unilateral decreases in the hippocampus [84]. The first study in MCI/AD using [11C]UCB-J compared Aβ+ patients (*n* = 10) with age-matched Aβ− controls and showed a significant reduction in hippocampal SV2A binding (41% decrease in BP_ND_) that survived partial volume correction and correlated significantly with episodic memory [89]. The decrease in SV2A binding throughout the neocortex in MCI/AD was modest and not significantly different from controls.

Multiple other candidates selective to SV2A have been developed including [18F]UCB-H, which displays a comparatively good signal but higher variability than [11C]UCB-J [90] and a human dosimetry of 19.7 mSv/MBq [91]. The longer [18F] half-life allows wider use and more time for transit to clinical/research sites [92].

Much future work involving SV2A imaging in AD remains. Currently, the first published AD study needs replication with more patients alongside longitudinal investigation. The relationship of SV2A binding with Aβ/tau needs to be explored as well as its relationship with other disease features such as mitochondrial dysfunction, cellular stress and glial (microglial and astrocyte) reactivity. A summary of discussed PET imaging can be found in Table 1.

## Magnetic Resonance Imaging

### Structural MR imaging

Structural imaging is the imaging workhorse of neurodegeneration, it is the most widely used and accessible, it is recommended in diagnostic guidelines [101] and it forms part of most consensus criteria. Structural MRI (sMRI) has several advantages over computed tomography (CT). Its main uses are (a) excluding brain lesions, (b) determining patterns of atrophy and (c) assessing vascular burden. Research key aims include improving early diagnosis and tracking disease progression.

#### Atrophy patterns—signatures of neurodegeneration

**Fig. 2:**
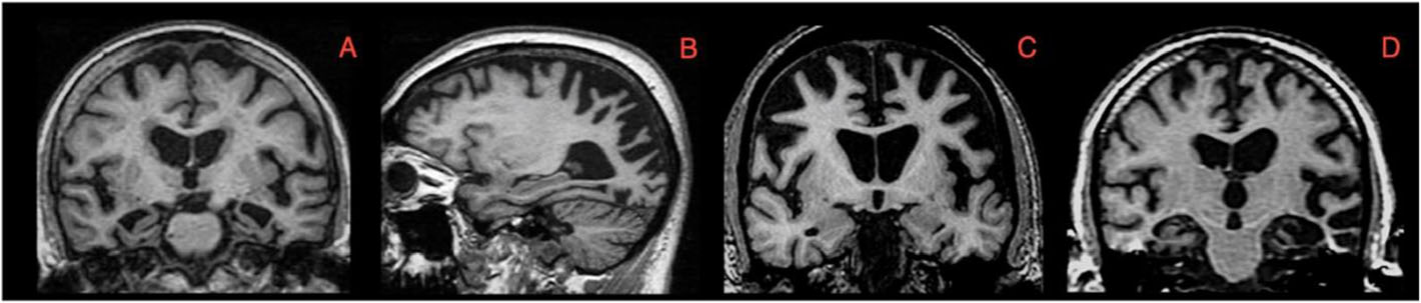
T1-weighted MRI scans demonstrating characteristic cortical atrophy signature in selected diseases: **a** typical amnestic Alzheimer’s disease, **b** posterior cortical atrophy, **c** behavioural variant frontotemporal dementia and **d** semantic dementia

Neurodegenerative disorders, to a greater or lesser extent, show characteristic patterns or signatures of brain atrophy on T1-weighted images (see Fig. 2) that can be used to improve differential diagnosis. Table 2 outlines some of the most common and useful atrophy patterns for the diagnosis of these diseases. Specific brain signatures have also been described in young onset AD [102, 103] and genetically mediated forms of frontotemporal dementia [104–106].

**Table 1:** Summary table of typical PET tracers for neurodegeneration-related investigations discussed in this article

Example tracers	Protocol	Analysis	Limitations
Glucose metabolism: [18F]FDG	• Fasting for ~ 4 h • Scanning 30 min after injection • Scan typically for 0–30 min	• SUV using weight and injected dose • SUVR using cerebellar grey matter or pons as reference regions [7]	• Hypometabolic patterns overlap between multiple neurodegenerative diseases [8–10] • Still not enough evidence to support routine clinical use in the prodromal phase [93]
Aβ: [11C]PiB [18F]Florbetaben [18F]Florbetapir [18F]Flutametamol [18F]NAV4694	Scan protocols vary between tracers; however, typically, patients are scanned 40–60 min (PiB) or 70–90 min (most [18F]-based tracers) after injection for ~ 20 min. For EANM clinical guidelines, see Minoshima et al. [94]	Typical analysis will use SUVR using the cerebellum or cerebellar grey matter as the reference region [21, 31, 32]	• [C11]PiB requires an on-site cyclotron • Second-generation tracers have certain off-target binding issues as well as reduced uptake in the cortex as compared to PiB [30] • Latest generation tracers have yet to be validated in larger cohorts • Aβ positivity can refer to various neurodegenerative diseases [95]
Tau: [18F]THK5351 [18F]THK5317 [18F]THK523 [11C]PBB3 [18F]Flortaucipir [18F]RO948 [18F]MK6240 [18F]GTP1 [18F]PI2620	Scan protocols vary between tracers; however, typically, patients are scanned in the range of 50–90 min after injection for ~ 20 min [96]	Most typical analyses will derive SUVR using the cerebellum, cerebellar grey matter or inferior cerebellum/cerebellar grey as the reference region [96].	• Molecular diversity of tauopathies means no single tau tracer can be used for all disorders [57] • First-generation tracers exhibit off-target binding and subcortical white matter uptake [96, 97] • Second-generation ligands have yet to be evaluated with regard to clinical outcomes in larger cohorts [54, 55, 96] • Experimental and clinical validation of tau tracers in general is still required [98, 99]
SV2A: [11C]UCB-J [18F]UCB-H	Scan protocols are yet to be determined in more studies using SV2A PET tracers	Centrum semi-ovale is most commonly used as the reference region, despite some evidence of synaptic changes [100]. Recently, also a cerebellar reference region has been suggested.	• Requires replication with more patients alongside longitudinal investigation [84, 89] • Association with other disease features (as described above) needs to be explored

It is also important to mention the utility of white matter hyperintensities (WMH) as these are essential for the diagnosis of cerebral small vessel disease (CSVD) [107]. Moreover, the location of microbleeds seen with T2*/SWI sequences can often bring diagnostic clarity on the underlying pathology—microbleeds associated with hypertension are found in deep brain regions, whereas Aβ-related microbleeds are more likely cortical [108]. Finally, diffusion-weighted imaging is the most sensitive sequence in the diagnosis of sporadic Creutzfeldt-Jakob disease [109].

**Table 2:** Atrophy patterns included in the current diagnostic criteria of selected neurodegenerative dementias. Only changes in T1-weighted MRI sequence are included in the MRI signature column. The MRI signatures described are supportive features for the diagnosis unless otherwise stated. *PPA* primary progressive aphasia. *FTD* frontotemporal dementia

Disease	Diagnostic criteria	MRI signature
Alzheimer’s disease	McKhann et al. [110]	Disproportionate atrophy in the medial, basal and lateral temporal lobe and medial parietal cortex
Posterior cortical atrophy	Crutch et al. [111]	Predominant occipito-parietal or occipito-temporal atrophy^a^
Logopenic variant PPA	Gorno-Tempini et al. [112]	Predominant left posterior perisylvian or parietal atrophy
Behavioural variant FTD	Rascovsky et al. [113]	Frontal and/or anterior temporal atrophy
Semantic variant PPA	Gorno-Tempini et al. [112]	Predominant anterior temporal lobe atrophy
Non-fluent variant PPA	Gorno-Tempini et al. [112]	Predominant left posterior fronto-insular atrophy
Dementia with Lewy bodies	McKeith et al. [114]	Relative preservation of the medial temporal lobe structures^b^
Multiple system atrophy	Gilman et al. [115]	Atrophy of the putamen, middle cerebellar peduncle, pons or cerebellum
Progressive supranuclear palsy	Höglinger et al. [116]	Atrophy predominant in the midbrain relative to pons

^a^Core neuroimaging feature of the PCA clinico-radiological syndrome; ^b^non-specific biomarker for DLB, but useful to differentiate from AD

#### Current use of structural MRI in research

Voxel-wise analyses confirm the value of brain atrophy patterns in pathologically distinct dementias [117–119]. In AD, volume loss appears later than Aβ deposition and synaptic dysfunction [120], but hippocampal changes are detected before symptoms [121]. Furthermore, hippocampal volume has been validated and accepted by regulatory agencies as a biomarker for trials targeting predementia stages [122]. Longitudinal rates of atrophy monitor progression and can change the sample size needed to show treatment effects depending on the technique and selected anatomical region [123, 124].

Cortical thickness has also been shown to be a marker of AD, where regionally specific cortical thinning can be used to detect presymptomatic Aβ-positive individuals but also can indicate the severity of symptoms [125, 126]. It has also been shown to be able to differentiate between neurodegenerative disorders, for example between AD and FTD [127]. Shape analysis can also be used in differentiating individuals, studies of hippocampal shape changes and atrophy have demonstrated differences in the substructural changes of the hippocampus depending on the type of neurodegenerative disorder [126, 128]. Shape analysis of brain ventricles has also shown that markers such as perimeters of the ventricles can be simple markers extracted from sMRIs to differentiate HC and AD [129].

#### Future directions of research

##### High-resolution volumetry of the medial temporal lobe in AD using 7T MRI

High spatial resolution sMRI now allows for volumetry of hippocampal subfields [130, 131]. Early changes in CA1 have been observed in AD, with volumetric studies indicating that CA1 atrophy measures may improve diagnostic accuracy at the MCI stage (see [120] for a review). Other studies, however, have found that the subiculum is associated with poorer cognitive performance and risk of developing dementia [132] and may serve as an early marker of AD-related atrophy [133]. Recent studies of volume, thickness and shape measurements of extrahippocampal subregions in the medial temporal lobe have shown that thickness measurements of the transentorhinal region could differentiate Aβ positive from negative individuals while outperforming other measures such as CA1 or whole hippocampal volume [134].

#### Assessment of iron deposition using in vivo MRI

Novel MRI techniques, such as quantitative susceptibility mapping (QSM) or the T2* transverse relaxation time, have shown that iron levels and its rate of accumulation are heterogeneous in the human brain [135] and correlates with cognitive impairment [136, 137] and slowing of motor performance [138, 139]. Abnormal iron deposition has been reported in AD [140, 141], Parkinson’s disease (PD) (for a review see [142]), multiple sclerosis [143, 144] and additional neurodegenerative disorders (for a review see [145]). The elevation in cortical iron deposition in PD is concordant with known alpha-synuclein pathology [146] and in AD, has been shown to predict cognitive decline in individuals with Aβ pathology [147].

Taken together, in vivo sMRI techniques may have the potential to improve early and differential diagnosis, aid stratification of patients into clinical trials and track disease progression in neurodegenerative disorders.

## Functional MRI, ASL, DTI and graph theory

### Functional MRI

**Fig. 3:**
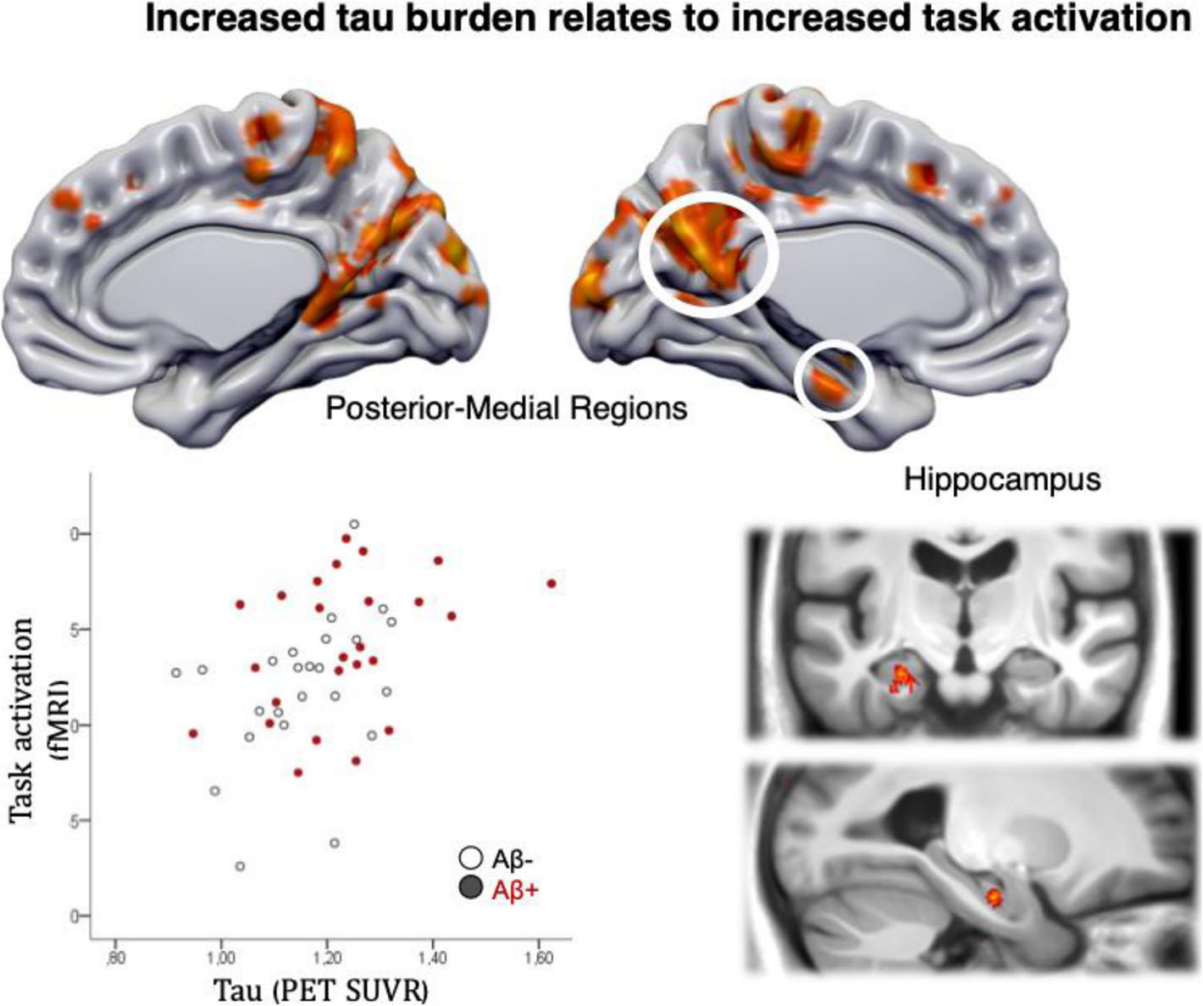
Cognitively normal older adults (*n* = 49) underwent 3-T fMRI while performing a mnemonic discrimination task as well as PET imaging. A whole-brain multiple regression showed that increased tau burden (mean flortaucipir SUVR from Braak III/IV ROI) was related to increased task activation during object processing (covarying for age and gender). Tau-related activation increases were seen mainly in hippocampus and posterior-medial regions. Results are FDR-corrected at the cluster level (*p*_cluster_ < .05, *p*_voxel_ < .001 uncorrected). The scatter plot (lower left) shows the correlation of regional Flortaucipir SUVR and object activation in posterior-medial regions. See Maass et al. [158] for study details

Neuronal dysfunction and altered connectivity of distinct brain networks are thought to occur early in the course of neurodegenerative diseases and can be measured indirectly with functional magnetic resonance imaging (fMRI). In AD, several resting-state fMRI studies revealed altered connectivity in the default mode network [148, 149], ranging from hippocampal coactivation [150] to potential compensatory increased activation in the MTL [151]. Studies have also suggested distinct atrophy patterns within various intrinsic functional networks for a number of neurodegenerative diseases [152]. Task-based fMRI studies are less consistent and have often reported increased task activation or reduced deactivation in hippocampus, frontal and parietal regions in the presence of AD pathology or in patients with MCI (e.g. [153–158]). There are also data indicating that an initial phase of hyperactivation [159] is followed by hypoactivation with further increasing Aβ burden and disease progression [160, 161]. Whether task-based fMRI shows hyper- or hypoactivation likely depends on the specific fMRI contrast/task, the brain region examined, and the pathological stage of an individual (for an example see Fig. 3). While task-based fMRI is a promising future biomarker, sensitivity and reliability of different fMRI tasks within-subject and across cohorts still need to be established.

### Arterial Spin Labelling

Current models of AD suggest that metabolic alterations occur in the brain before structural changes could be identified. FDG PET (as discussed earlier) has been a standard tool for measuring these changes in the past; however, due to the introduction of tau and Aβ-specific PET tracers (as discussed previously), there is increased demand for a biomarker that does not require a second PET scan. Arterial spin labelling (ASL) has shown to be a promising replacement for FDG PET; this is due to the metabolism and perfusion in the brain being very closely matched [162] and so hypometabolic patterns seen in FDG PET can be equally seen in ASL images. This fact coupled with patients already undergoing an MRI scan means that ASL could serve as a cheaper and faster alternative which would also reduce the radiation burden to the patient without sacrificing any diagnostic quality for both AD and FTD [163, 164]. For both AD and other neurodegenerative diseases, there is still a requirement for larger studies to validate this technique [163, 165].

### DTI

The brain’s white matter tracts are also sensitive to the underlying pathology of neurodegenerative disease [166, 167]. Using diffusion tensor imaging (DTI), a variant of MRI that is sensitive to the mobility of water molecules in tissue, it is possible to quantify the microstructural properties of white matter tracts in vivo [168]. In AD, DTI studies have identified microstructural alterations (specifically increased absolute diffusivities and reduced fractional anisotropy) in tracts linking regions affected early by disease pathology, including the fornix, parahippocampal cingulum and corpus callosum [169–171]. Microstructural variation in these tracts has also been associated with the accumulation of Aβ and tau in cognitively normal individuals [80, 172], suggesting that DTI may prove useful as a biomarker for AD. Similarly, alterations in diffusivity have been described in presymptomatic and early-stage familial frontotemporal dementia. Individuals suffering from amyotrophic lateral sclerosis (ALS) were also seen to have increased diffusivity in the bilateral centrum semi-ovale as well as deep and parietal white matter [173]. However, common DTI measures such as fractional anisotropy are influenced by numerous disease-relevant properties, including myelination, thereby limiting anatomical specificity [174]. Thus, while DTI has proven to be useful as a tool for understanding neurodegenerative conditions, further research is needed to establish its utility as a biomarker.

### Graph theory

Graph theory is the study of systems of interactive elements—‘nodes’, and the connections between them—‘edges’ [175, 176], allowing for representation of brain networks. Both structural (DTI) and functional connectivity measures (fMRI) can be obtained from brain graphs [177, 178]. AD research uses graph theory to examine integration (path length between nodes), segregation (clustering) and centrality (importance of nodes in a network) [179]. Network topology appears to be disrupted, with clinical symptoms arising from changes in communication between anatomically and functionally connected brain areas [179]. AD appears to result in longer paths between nodes with lower global efficiency [179] and with less interconnectivity and more segregated clusters in the default mode network (DMN) [180]. Widespread changes within and outside the DMN are seen with advanced Aβ accumulation [181]. Inconsistent findings between AD studies may be due to different definitions of nodes and edges [182]. Harmonisation is needed for future work.

## Machine learning

Current reviews of ML algorithms applied to neurodegenerative disorders include a systematic review of the use of ML and neuroimaging in general to assist the diagnosis of dementia [183, 184] to more methodological reviews, focusing on feature extraction, different ML architectures and validation techniques [185–187]. Three longitudinal studies analysed the progression of AD [188–190] using structural MRI and deep learning (DL) algorithms such as recurrent neural networks (RNNs) and variations of long short-term memory networks (LSTMN). The most common feature in order to study disease progression is hippocampal volume.

### Early diagnosis and progression to MCI/AD

The majority of ML studies are focused on diagnosis or early detection of AD [191–213]. There has been an increasing effort to try to fully predict AD from MCI or healthy controls as well as using artificial intelligence techniques (such as ML or DL) in order to aid clinical diagnosis. Lately, more importance has been given to subject memory complaints (SMC) as it could be a pre-asymptomatic stage of MCI.

There are several longitudinal databases that are helping to develop these kinds of studies such as ADNI, OASIS or the Rotterdam Study. With this increase of data, there has been a shift from the use of ML algorithms such as support vector machines (SVMs) and k nearest neighbours (KNN) to more DL-based studies, mostly convolutional neural networks (CNNS) [205, 214, 215]. Along with feature selection methods, these models combine different sMRI cortical and subcortical volumetric measures to identify disease subtypes [216]. Neural networks (NNs) based on sMRI and cognitive scores can predict the conversion of MCI to AD (cMCI) and distinguish between stable MCI and cMCI [214, 217, 218]. ML classifiers can also differentiate between clinical syndromes of frontotemporal dementia (FTD) [219]. Longitudinal studies using feature extraction-based learning techniques provide improved atrophy measures with significantly lower mean absolute error and volumetric markers such as the hippocampus, posterior cingulate cortex and middle temporal gyrus for evaluating disease progression in AD and MCI [190, 220, 221].

### Multimodal machine learning

ML is an optimal approach to combine the findings of different imaging modalities. NNs based on grey matter density from MRI and glucose metabolism from PET yields better results than individual modalities [215, 222, 223]. Structural and connectivity measures from MRI combined with metabolism rate from PET predict the conversion of MCI to AD. Deep learning models can predict cMCI from non-white matter extractions using PET images combined with MRI images. NNs based on sMRI or resting-state fMRI, cognitive and functional assessments show enhanced automatic diagnosis of both AD and MCI [224]. ML techniques can be used to combine clinical measures with multiple imaging modalities to understand the neuropathological processes of diseases [225].

## Conclusion

**Fig. 4:**
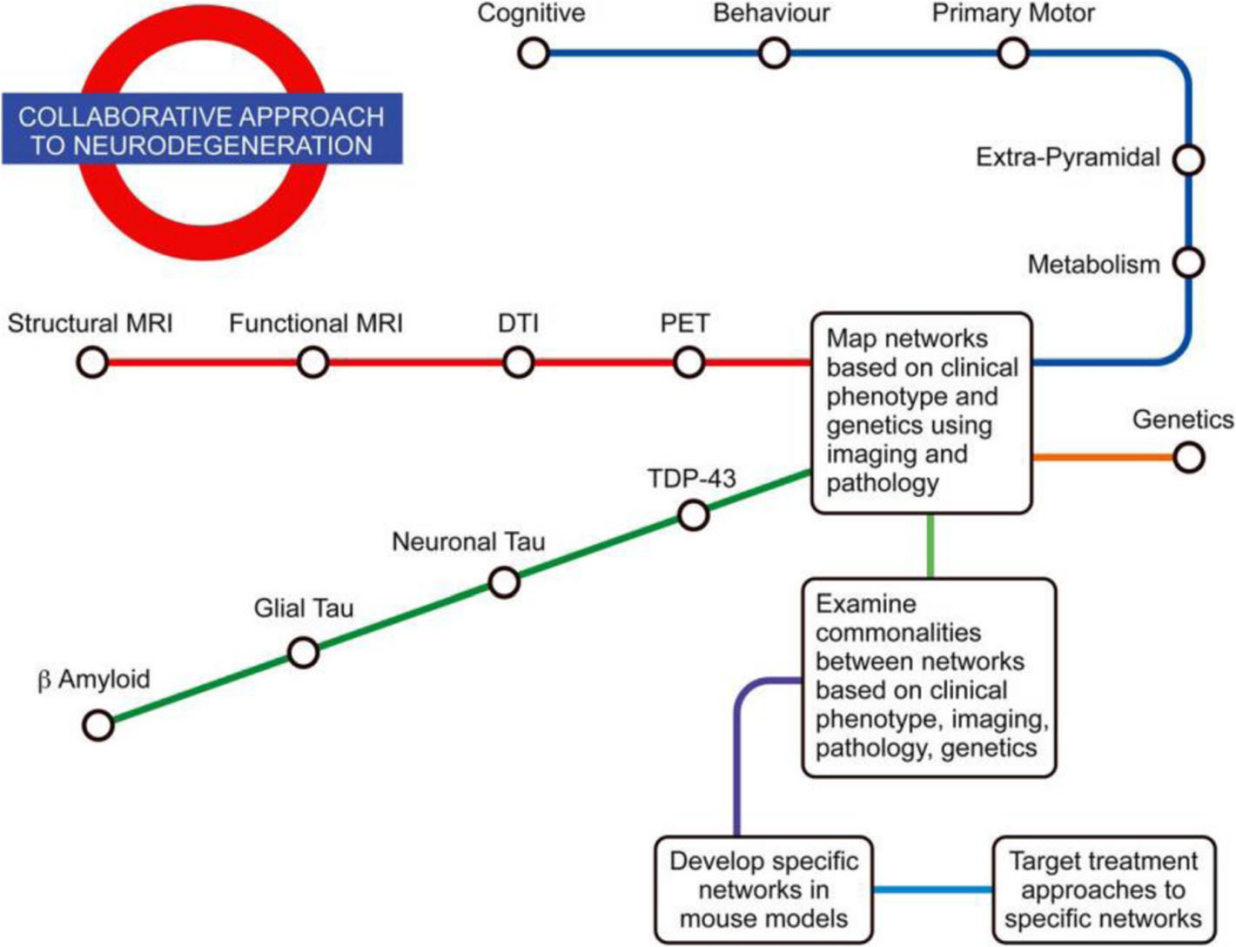
The imaging arm (red) as part of the greater collaborative approach to neurodegeneration [226]

There is a growing body of evidence that imaging biomarkers can be useful in the detection and monitoring of neurodegenerative diseases. Due to the complexity of many of the diseases being studied as well as a variation in the results reported, extracting definitive findings remains a challenge. With ongoing and planned trials for various treatments, it is important to incorporate imaging biomarkers into these trials as well as continuing to improve the diagnostic and prognostic power of these techniques. On a wider scale, imaging biomarkers have a part to play in a collaborative approach to neurodegeneration (Fig. 4) as understanding and treatment becomes increasingly multidisciplinary.

## Data Availability

This review does not contain any analysable data. All sources cited in this paper are publicly available.

## Abbreviations

1TC: 1 tissue compartment (model)
AD: Alzheimer’s disease
ASL: Arterial spin labelling
Aβ: Amyloid-beta
cMCI: Converting mild cognitive impairment
CSF: Cerebrospinal fluid
CSVD: Cerebral small vessel disease
CT: Computed tomography
DLB: Dementia with Lewy bodies
DMN: Default mode network
DTI: Diffusion tensor imaging
FDG: 2-Fluoro-2-deoxy-d-glucose
fMRI: Functional magnetic resonance imaging
FTD: Frontotemporal dementia
KNN: k nearest neighbour
LSTMN: Long short-term memory network
MCI: Mild cognitive impairment
MRI: Magnetic resonance imaging
NFT: Neurofibrillary tangle
NN: Neural network
PD: Parkinson’s disease
PET: Positron emission tomography
PHF: Paired helical filament
PiB: Pittsburgh compound B
QSM: Quantitative susceptibility mapping
RNN: Recurrent neural network
SMC: Subject memory complaints
sMRI: Structural magnetic resonance imaging
SRTM: Simplified reference tissue model
SUV: Standardised uptake value
SUVR: Standardised uptake value ratio
SV2A: Synaptic vesicle glycoprotein 2A
SVM: Support vector machines
WMH: White matter hypointensities
